# A Primed Subpopulation of Bacteria Enables Rapid Expression of the Type 3 Secretion System in Pseudomonas aeruginosa

**DOI:** 10.1128/mBio.00831-21

**Published:** 2021-06-22

**Authors:** Christina K. Lin, Daniel S. W. Lee, Saria McKeithen-Mead, Thierry Emonet, Barbara Kazmierczak

**Affiliations:** a Medical Scientist Training Program, Yale University, New Haven, Connecticut, USA; b Department of Molecular Biophysics and Biochemistry, Yale University, New Haven, Connecticut, USA; c Department of Medicine (Infectious Diseases), Yale University, New Haven, Connecticut, USA; d Department of Molecular, Cellular, and Developmental Biology, Yale University, New Haven, Connecticut, USA; e Department of Physics, Yale University, New Haven, Connecticut, USA; f Department of Microbial Pathogenesis, Yale University, New Haven, Connecticut, USA; University of Pittsburgh School of Medicine

**Keywords:** *Pseudomonas aeruginosa*, type 3 secretion system, gene regulation, lineage tracking, pathogenesis, phenotypic heterogeneity

## Abstract

Type 3 secretion systems (T3SS) are complex nanomachines that span the cell envelope and play a central role in the biology of Gram-negative pathogens and symbionts. In Pseudomonas aeruginosa, T3SS expression is strongly associated with human disease severity and with mortality in murine acute pneumonia models. Uniform exposure of isogenic cells to T3SS-activating signal results in heterogeneous expression of this critical virulence trait. To understand the function of such diversity, we measured the production of the T3SS master regulator ExsA and the expression of T3SS genes using fluorescent reporters. We found that heterogeneous expression of ExsA in the absence of activating signal generates a “primed” subpopulation of cells that can rapidly induce T3SS gene expression in response to signal. T3SS expression is accompanied by a reproductive trade-off as measured by increased division time of T3SS-expressing cells. Although T3SS-primed cells are a minority of the population, they compose the majority of T3SS-expressing cells for several hours following activation. The primed state therefore allows P. aeruginosa to maximize reproductive fitness while maintaining the capacity to quickly express the T3SS. As T3SS effectors can serve as shared public goods for nonproducing cells, this division of labor benefits the population as a whole.

## INTRODUCTION

Clonal bacterial populations exhibit phenotypic heterogeneity (1, 2). Such variability can allow populations to “bet-hedge” in the setting of unpredictable environmental variation (3) or create a “division of labor” that enables cooperative behaviors between phenotypically diverse individuals (4). Phenotypic variation among pathogenic bacteria can lead to “cooperative virulence,” with subsets of individuals producing goods (e.g., toxins, proinflammatory molecules) that provide benefit to the group at substantial individual cost (5, 6). One well-described example of cooperative virulence is provided by Salmonella enterica serovar Typhimurium (*S.* Typhimurium), a gastrointestinal pathogen whose Salmonella pathogenicity island 1 (SPI-1) type 3 secretion system (T3SS) elicits a proinflammatory host response that is necessary for *S*. Typhimurium to overcome colonization resistance and establish infection in the gut (7). Because cooperative behaviors can be exploited by mutant “cheaters,” who benefit from shared goods without incurring production costs, there is significant interest in understanding how cooperative behaviors resist destabilization by the emergence of fast-growing cheaters. For *S*. Typhimurium, the expression of SPI-1 by only a subpopulation of individuals during infection appears key (5, 8). Genetically wild-type but phenotypically nonexpressing individuals can both compete with SPI-1 mutants that might emerge during infection and maintain a population of individuals capable of SPI-1 expression that is necessary for systemic infection and transmission (9).

Pseudomonas aeruginosa is a human opportunistic pathogen whose expression of a T3SS is significantly correlated with increased risk of acute infection, poor clinical outcomes, and mortality in human and murine respiratory tract infections (10–12). Like *S.* Typhimurium, clonal populations of P. aeruginosa bacteria are observed to express T3SS proteins heterogeneously (13–18). Substrates of the T3SS include two bifunctional Rho-family GTPase activating proteins (GAPs)/ADP ribosyl transferases (ADPRTs), ExoS and ExoT; a phospholipase A_2_, ExoU; and a nucleotidyl cyclase, ExoY (19–21). The ExoU cytotoxin, whose rapid production during acute respiratory tract infection protects P. aeruginosa from neutrophil-mediated clearance (22), can function as a shared public good during infection. Otherwise avirulent T3SS-deficient P. aeruginosa can outcompete coinfecting T3SS-expressing cells during acute infection of the murine lung, benefiting in *trans* from ExoU-mediated killing of recruited neutrophils (14).

**FIG 1:**
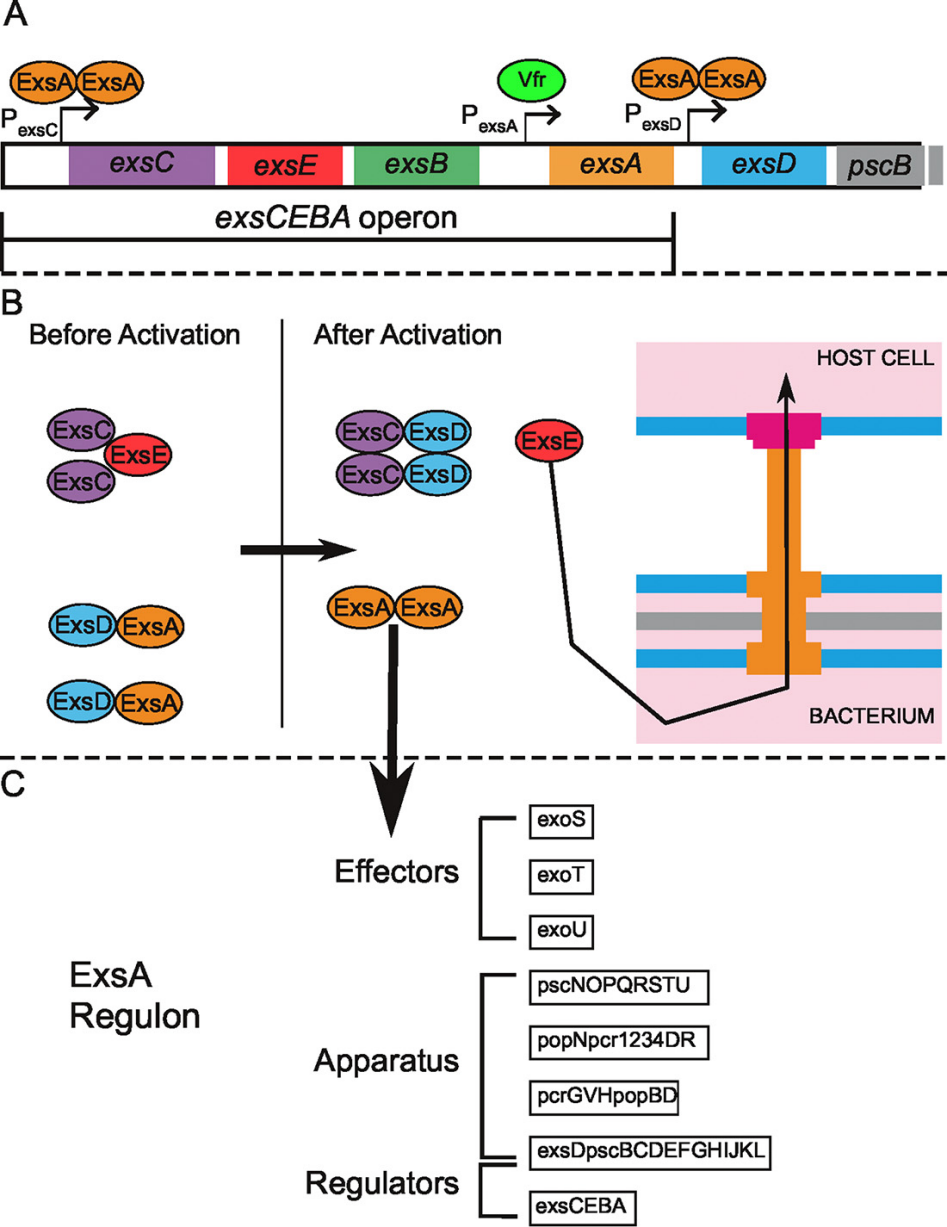
The T3SS regulatory cascade. (A) The T3SS master regulator ExsA positively regulates its own transcription by binding to the P_exsC_ promoter, which makes the polycistronic *exsCEBA* transcript. ExsA also upregulates the expression of its own antiactivator, ExsD. A second promoter, P_exsA_, drives expression of *exsA* alone and is under positive regulation by Vfr-cAMP. (B) The *exsECBA* operon encodes regulators that link T3SS gene expression to activation of type 3 secretion. Prior to activation, ExsE sequesters ExsC, and ExsD binds to and inhibits ExsA from homodimerizing and acting on its target promoters. When type 3 secretion is triggered, ExsE is secreted via the T3SS, releasing ExsC. ExsD is then sequestered by ExsC, and ExsA is free to act on its target promoters and fully upregulate T3SS expression. Note that an intact and functional T3SS apparatus must be present in order to activate the cascade and upregulate T3SS expression. (C) ExsA dimerizes and binds to target promoters in its regulon, upregulating expression of T3SS apparatus genes and effector proteins in addition to the *exsCEBA* operon.

Although we do not know how heterogeneous T3SS expression is established in P. aeruginosa, much is known about the transcriptional regulation of T3SS genes. ExsA, a LysR/AraC family transcriptional activator, is required for T3SS gene expression; the many convergent signals and pathways that regulate ExsA transcription, translation, and protein activity have been recently reviewed (23). Binding of ExsA homodimers to target promoters upregulates the expression of the 10 transcriptional units that encode ∼40 structural, regulatory, and effector proteins of the T3SS (24–26). ExsA positively regulates its own expression by binding to the promoter of the *exsCEBA* operon (P_exsC_) (Fig. 1A); a second promoter (P_exsA_) located in the intergenic region between *exsB* and *exsA* is controlled by the cAMP-dependent transcription factor Vfr and generates a transcript encoding only ExsA (16, 27). ExsD, an antiactivator of ExsA, is encoded within a separate operon immediately downstream of *exsCEBA* that is also regulated by ExsA (28).

The ExsA, ExsD, ExsC, and ExsE proteins control T3SS expression in response to activating cues, such as the divalent cation chelator nitrilotriacetic acid (NTA). In the absence of these cues, ExsD sequesters ExsA and prevents DNA binding (28); ExsC and ExsE likewise form a complex. When T3SS-activating signals or host cell contact causes the T3SS apparatus to open, ExsE is secreted, allowing ExsC to bind ExsD (Fig. 1B) (18, 29). This in turn frees ExsA to dimerize and act on its target promoters (18, 30) (Fig. 1C). The dual positive feedback loops present in this regulatory circuit—ExsA promotes transcription of ExsC (an inhibitor of ExsD activity) as well as of itself—combine to form a highly robust bistable switch that has been characterized in the heterologous host Escherichia coli (31).

In this study, we examined how the unique regulatory cascade governing T3SS expression in P. aeruginosa leads to phenotypic heterogeneity under uniform T3SS-activating conditions *in vitro*. By simultaneously tracking expression of T3SS genes and the positive regulator ExsA at the single-cell level, we describe when heterogeneity is established and how it affects fitness and virulence of P. aeruginosa.

## RESULTS

### P. aeruginosa exhibits phenotypic heterogeneity of T3SS expression during planktonic growth in T3SS-activating conditions.

**FIG 2:**
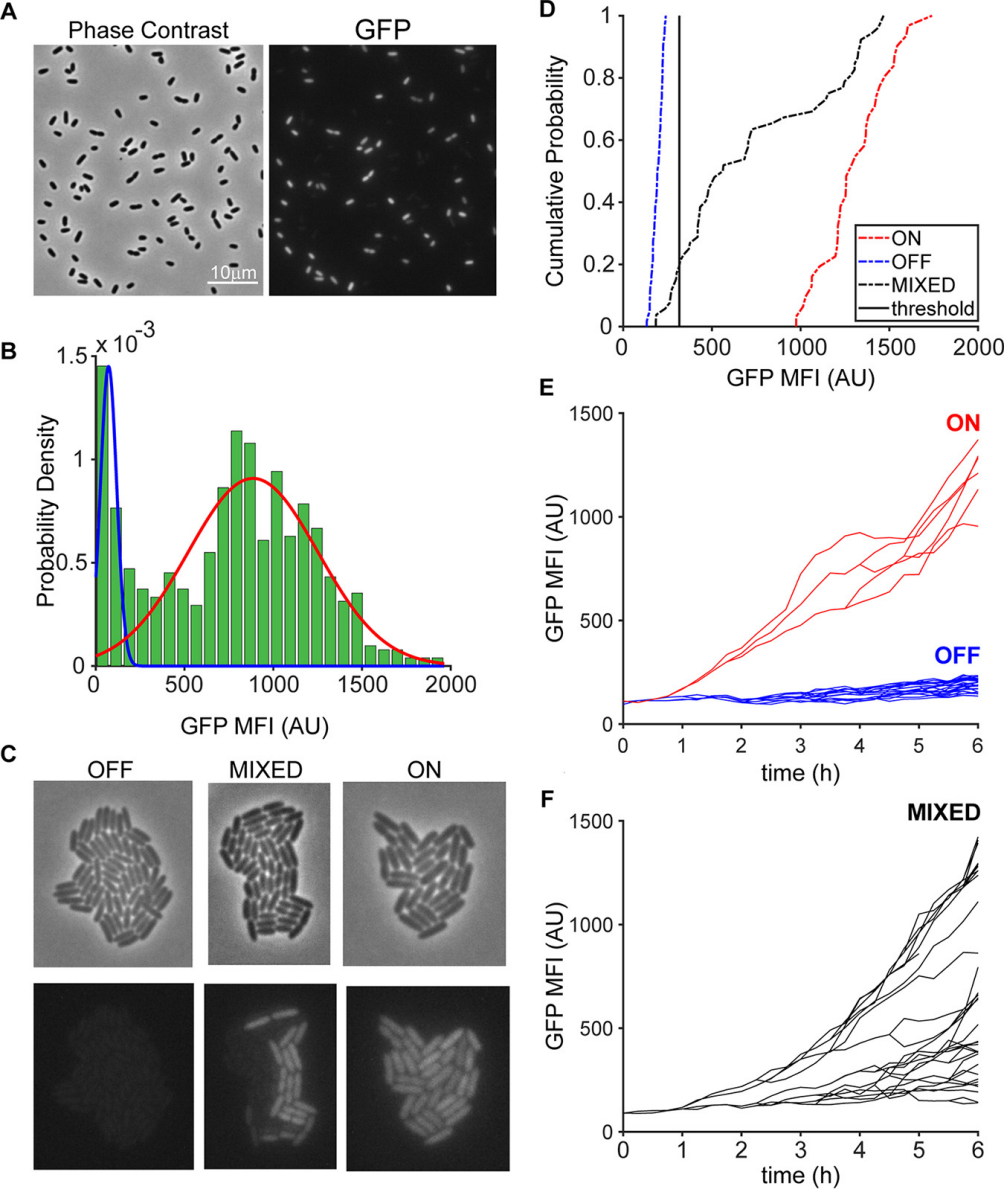
P. aeruginosa expresses T3SS genes heterogeneously in response to activating signal. (A) Both T3SS-ON and T3SS-OFF cells are present after growth of PA14 P*_exoT_*-sfGFP for 8 h in T3SS-activating medium (MinS + 10 mM NTA) under planktonic conditions. (B) A mixed Gaussian model fit to GFP mean fluorescence intensity (MFI) of PA14 P_exoT_-sfGFP cells is consistent with a bimodal population (*n* = 2,292 cells). (C) Phase and GFP fluorescence still images at *t* = 6 h from representative ON, OFF, and mixed microcolony time-lapse movies. (D) Cumulative probability distribution of GFP MFI at *t* = 6 h is graphed for microcolonies in panel C. The threshold (black line) defines the decision boundary between T3SS-ON and T3SS-OFF as defined by positive (Δ*exsD*) and negative (Δ*exsA*) controls. (E and F) Three types of microcolonies are identified by single-cell lineage tracing of PA14 P_exoT_-sfGFP microcolonies arising from single cells grown for 6 h on agarose pads containing activating signal. The lineage traces shown correspond to colonies in panel C as follows: ON and OFF in panel E and mixed in panel F.

Transcription of the gene encoding the ExoT T3SS effector is strongly induced when P. aeruginosa is exposed to tissue culture cells or medium containing a divalent chelator, such as MinS+NTA (MinS medium plus 10 mM NTA) (32, 33). We took advantage of this to construct a sensitive T3SS transcriptional reporter coupled to superfolder green fluorescent protein (GFP) (P_exoT_-sfGFP) expression, which was integrated into the chromosomal *attB* site of PA14 wild-type, Δ*exsA*, and Δ*exsD* bacteria (Fig. S1). Expression of sfGFP in wild-type bacteria required growth in T3SS-activating medium, as no fluorescence was observed when cells were cultured in MinS+Ca (MinS medium plus 5 mM CaCl_2_) for ≥8 h (Fig. S1A). sfGFP expression was not observed in Δ*exsA* bacteria grown in T3SS-activating medium (MinS+NTA) (Fig. S1B), while Δ*exsD* cells were uniformly fluorescent under both T3SS-activating and -nonactivating (MinS+Ca) conditions (Fig. S1C). Both fluorescent and nonfluorescent cells were observed throughout an 8-h experiment when wild-type bacteria were cultured under T3SS-activating conditions and sampled for microscopy every 2 h (Fig. 2A). A mixed Gaussian could be fitted to the histogram of GFP mean fluorescence intensity (Fig. 2B), consistent with the presence of a bimodal population of phenotypically T3SS-expressing and -nonexpressing bacteria. The presence of both GFP-negative and -positive bacteria under T3SS-activating growth conditions was likewise observed using a second reporter that coupled sfGFP expression to the *exoU* promoter, P_exoU_-sfGFP, in three additional lab strains (PAO1, PAK, and PA103) (Fig. S2).

10.1128/mBio.00831-21.3 FIG S1 The P*_exoT_*-sfGFP reporter responds to extrinsic and intrinsic T3SS-activating signals. (A) PA14 WT, (B) PA14 Δ*exsA*, and (C) PA14 Δ*exsD* with the *attB*::P_exoT_-sfGFP reporter were grown in MinS + 10 mM NTA (T3SS-inducing conditions) or MinS + 5 mM CaCl_2_ (T3SS-noninducing conditions) as indicated. Download FIG S1, PDF file, 1.6 MB. Copyright © 2021 Lin et al. 2021 Lin et al. https://creativecommons.org/licenses/by/4.0/ This content is distributed under the terms of the Creative Commons Attribution 4.0 International license.

10.1128/mBio.00831-21.4 FIG S2 Heterogeneous T3SS is observed in multiple P. aeruginosa strain backgrounds. P. aeruginosa strains PAO1, PAK, PA14, and PA103 with the *attB*::P_exoU_-sfGFP reporter were grown in MinS + 10 mM NTA, sampled at the indicated times and analyzed by flow cytometry. PA103Δ*exsA* and PA103Δ*exsD* serve as negative and positive controls, respectively. Download FIG S2, PDF file, 0.1 MB. Copyright © 2021 Lin et al. 2021 Lin et al. https://creativecommons.org/licenses/by/4.0/ This content is distributed under the terms of the Creative Commons Attribution 4.0 International license.

These experiments demonstrated that both T3SS-expressing and -nonexpressing bacteria were present within a clonal population exposed to uniform T3SS-activating signals but gave no insight about an individual cell’s history or trajectory of T3SS expression. We therefore developed a time-lapse method to trace the fate of individual cells inoculated from non-T3SS-activating medium onto an agarose pad that contained the activating signal. Both phase and fluorescence images of cells were obtained every 15 min over a period of 6 to 8 h. Single wild-type cells (PA14 P_exoT_-sfGFP) gave rise to three types of microcolonies, in which daughter cells were all brightly fluorescent, all dimly fluorescent, or consisted of a mixture of both GFP-bright and GFP-dim cells at the end of a 6- to 8-h time-lapse imaging series (Fig. 2C, Movie S1). We used the control strains Δ*exsA* P_exoT_-sfGFP (T3SS-negative) and Δ*exsD* P_exoT_-sfGFP (constitutively T3SS-positive) to define a fluorescence threshold at which cells had a 50% chance of being T3SS-positive. When wild-type cells crossed this threshold during a time-lapse experiment, they were classified as “T3SS-ON”; if their fluorescence was below this threshold, they were classified as “T3SS-OFF” (Fig. S3). Microcolonies were scored according to the proportion of T3SS-ON cells they had at the end of a 6-h time-lapse experiment; colonies with >95% T3SS-ON cells were scored as ON, those with <5% T3SS-ON cells were scored as OFF, and all others with an intermediate proportion of T3SS-ON cells were scored as mixed (Fig. 2D).

10.1128/mBio.00831-21.5 FIG S3 A T3SS-ON threshold is defined using constitutively expressing and nonexpressing strains. (A) To define individual cells as either T3SS-ON or T3SS-OFF, positive (PA14 Δ*exsD*) and negative (PA14 Δ*exsA*) controls with the P_exoT_-sfGFP reporter were grown under the T3SS-inducing conditions and tracked with time-lapse microscopy. A decision threshold was calculated using the intensity distributions of the positive and negative controls. Each distribution was fit to a Gaussian, and the threshold was defined as the intensity at which the likelihood of each Gaussian was equal, i.e., where the log likelihood ratio of the MLE-fit Gaussians was equal to 0. Equivalently, a *P* value could be calculated since the statistic −2 · log(K) (K = likelihood ratio) is drawn from a chi-squared distribution and the threshold was calculated where *P* = 0.5 and lower and upper confidence intervals were set where *P* = 0.05 and 0.95, respectively. A cell with a specific value of GFP MFI above the threshold has a higher likelihood of being T3SS-ON. The decision threshold at hour 6 is shown. (B) The decision threshold (in purple) graphed over 8 h, with the GFP mean fluorescence intensity ranges for the positive (PA14 Δ*exsD*) and negative (PA14 Δ*exsA*) controls shown in black and green, respectively. Download FIG S3, PDF file, 0.3 MB. Copyright © 2021 Lin et al. 2021 Lin et al. https://creativecommons.org/licenses/by/4.0/ This content is distributed under the terms of the Creative Commons Attribution 4.0 International license.

10.1128/mBio.00831-21.8 MOVIE S1 Time-lapse microscopy movies of PA14 WT P_exoT_-sfGFP grown in T3SS-activating (MinS + 10 mM NTA) agarose pads at 37°C for up to 6 to 8 h. Both the phase contrast and GFP channels are shown, with 15 min between each frame, for the ON, mixed, and OFF microcolonies depicted in Fig. 1. Download Movie S1, AVI file, 5 MB. Copyright © 2021 Lin et al. 2021 Lin et al. https://creativecommons.org/licenses/by/4.0/ This content is distributed under the terms of the Creative Commons Attribution 4.0 International license.

By tracking GFP mean fluorescence intensity (MFI) for individual cells and their progeny over time, we observed distinct trajectories of T3SS expression for ON, OFF, and mixed microcolonies (Fig. 2E). Overall, cells for which we measured GFP fluorescence above background demonstrated a monotonic increase in GFP MFI over time. A small number of cells quickly became GFP-positive when exposed to T3SS-activating conditions, and their daughters maintained this phenotype; these founder cells gave rise to ON microcolonies (Fig. 2E, red lineage tracing). Cells within mixed microcolonies showed delayed expression of GFP; T3SS-ON cells arose throughout the time-lapse imaging experiments and were observed to yield T3SS-ON daughters. Lastly, OFF colonies arose from T3SS-OFF cells whose progeny remained nonfluorescent despite exposure to T3SS-activating conditions. The trajectory of ON colonies, which appeared to arise from cells uniquely capable of an immediate response to T3SS-activating signals, prompted us to examine whether the expression of T3SS genes or proteins differed among cells before they were exposed to T3SS-activating conditions.

### ON microcolonies are derived from “primed” mother cells.

**FIG 3:**
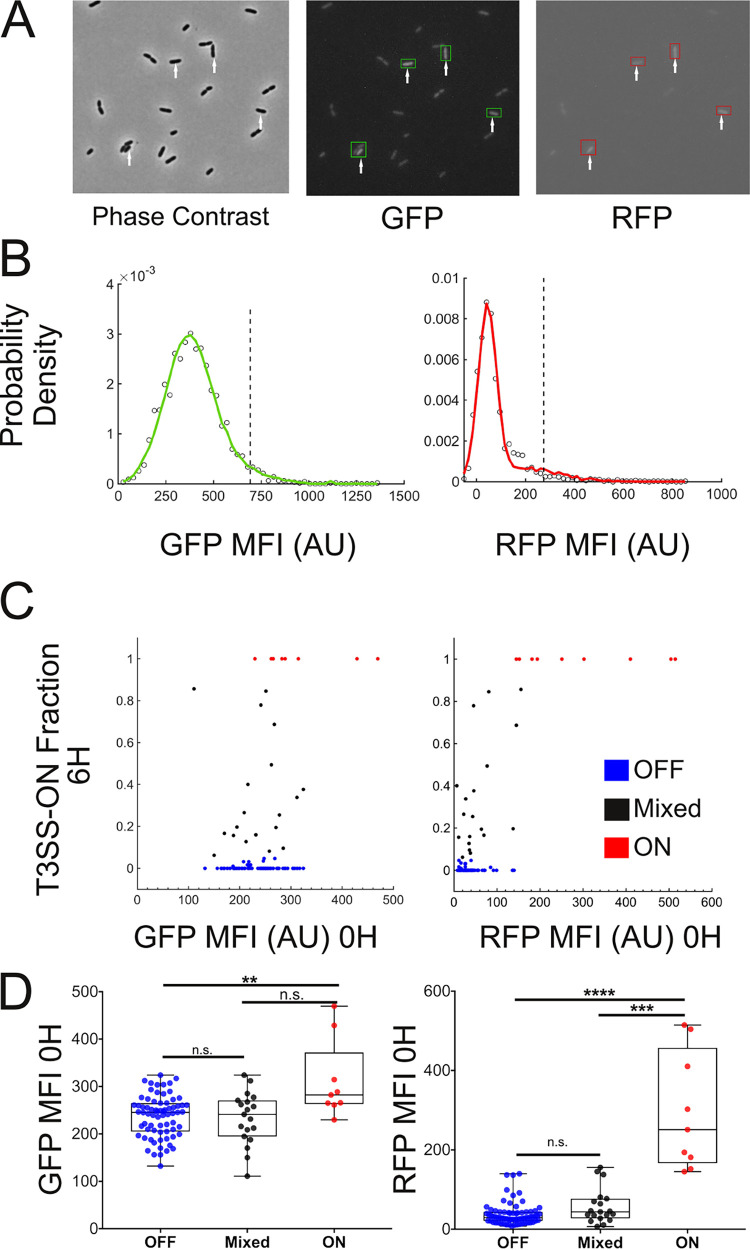
“ON” microcolonies descend from “primed” cells. (A) PA14 dual reporter P_exoT_-sfGFP/*exsA-mTagRFP-t* were grown in T3SS-nonactivating conditions for 4 h prior to imaging at the time of transfer (*t* = 0 h) to agarose pads containing activating signal. White arrows indicate individual cells with greater GFP and RFP MFI than the population average. (B) Distributions of GFP and RFP MFI at *t* = 0 h; dashed black vertical lines indicate an MFI value two standard deviations (SD) above the population mean (*n* = 2,435 cells). The mean GFP MFI was 394 AU (median, 383.5) with an SD of 149.4. The mean RFP MFI was 79.5 AU (median 53.6) with an SD of 97.8. The proportion of cells with GFP or RFP MFIs more than 2 SD above the mean was 3.6% and 4.4%, respectively. (C) GFP and RFP MFIs of PA14 P_exoT_-sfGFP/*exsA-mTagRFP-t* mother cells at *t* = 0 h versus the proportion of T3SS-ON cells at *t* = 6 h in the derived microcolony (*n* = 99). (D) Box and whisker plots show the GFP and RFP MFI (*t* = 0 h) of individual mother cells yielding ON, OFF, and mixed microcolonies. MFIs were compared between groups using the Kolmogorov-Smirnov test. The RFP MFI of ON mother cells differed significantly from that measured for cells giving rise to either Mixed (*P* = 0.0001) or OFF microcolonies (*P* < 0.0001); the GFP MFI differed significantly only between ON versus OFF mother cells (*P* = 0.0032).

The regulatory cascade that leads to T3SS gene expression begins with the secretion of ExsE through the T3SS, which frees ExsC to sequester ExsD and thereby relieve ExsA repression (18, 30). This mechanism, however, implies that a cell must already have a functional T3SS apparatus in order to respond to T3SS-activating signals. We hypothesized that individual bacteria whose sfGFP fluorescence rapidly increased might be such cells, “primed” to respond to activating signals by virtue of prior ExsA expression and T3SS apparatus assembly. We therefore constructed a second reporter to measure ExsA expression by inserting an ribosome binding site (RBS)-*mTagRFP-t* cassette into the chromosome immediately after the *exsA* gene. PA14 carrying both P_exoT_-sfGFP and *exsA-mTagRFP-t* reporters (“dual reporter”) was imaged under T3SS-nonactivating conditions, using longer exposure times to capture an extended dynamic range of fluorescence signals. The distributions of red fluorescent protein (RFP) and GFP MFI in these cells were skewed; we observed a tail of RFP-bright cells under these conditions, as well as some cells with increased GFP fluorescence (Fig. 3A and B). When we carried out time-lapse imaging of the PA14 dual reporter strain under these calcium-replete (T3SS nonactivating) conditions, we observed that all microcolonies remained GFP-dim (i.e., below the T3SS-ON threshold) after 6 h of induction, but some contained RFP-bright bacteria (Movie S2). Thus, it appeared that some cells had expressed ExsA even in the absence of T3SS-activating signals.

10.1128/mBio.00831-21.9 MOVIE S2 Time-lapse microscopy movies of PA14 WT P_exoT_-sfGFP/*exsA-mTagRFP-t* grown in T3SS-activating (MinS + 10 mM NTA) agarose pads at 37°C for 6 to 8 h. The phase contrast, RFP, and GFP channels are shown for an ON, mixed, and OFF microcolony, in sequence. Download Movie S2, AVI file, 0.3 MB. Copyright © 2021 Lin et al. 2021 Lin et al. https://creativecommons.org/licenses/by/4.0/ This content is distributed under the terms of the Creative Commons Attribution 4.0 International license.

We next carried out time-lapse imaging of the dual-reporter strain, tracking individual “mother” cells inoculated from nonactivating cultures onto agar pads containing T3SS-activating signals (Movie S3). We again observed that single cells gave rise to ON, OFF, or mixed microcolonies, as defined above by the proportion of T3SS-ON (GFP+) cells. We plotted the RFP and GFP MFI of each mother cell at time 0 (intensity [*t* = 0 h]) against the proportion of T3SS-ON cells in the microcolony derived from that cell at hour 6 (Fig. 3C and D). The initial RFP MFI of mother cells that gave rise to ON colonies was significantly greater than that measured for mother cells which gave rise to mixed or OFF colonies (Fig. 3D); the initial GFP MFI of these mother cells also differed from those yielding mixed or OFF colonies. Mother cells that gave rise to mixed versus OFF colonies, however, had initial RFP and GFP MFI that were indistinguishable from each other. In aggregate, these observations suggested that a subpopulation of P. aeruginosa cells express ExsA in the absence of T3SS-activating signals and that these cells are “primed” to respond rapidly to T3SS-activating signals by upregulating the transcription of T3SS genes.

10.1128/mBio.00831-21.10 MOVIE S3 Time-lapse microscopy movie of PA14 WT P_exoT_-sfGFP/*exsA-mTagRFP-t* grown in T3SS-nonactivating (MinS + 5 mM CaCl_2_) agarose pads at 37°C for 8 h. Both the RFP and GFP channels are shown, with 15 min between each frame. Some microcolonies, like the one shown, displayed RFP-positive cells, though GFP-positive cells were never observed under this condition. Download Movie S3, AVI file, 5.8 MB. Copyright © 2021 Lin et al. 2021 Lin et al. https://creativecommons.org/licenses/by/4.0/ This content is distributed under the terms of the Creative Commons Attribution 4.0 International license.

### ExsA expression in individual cells is temporally linked with the switch from the T3SS-OFF to T3SS-ON state.

**FIG 4:**
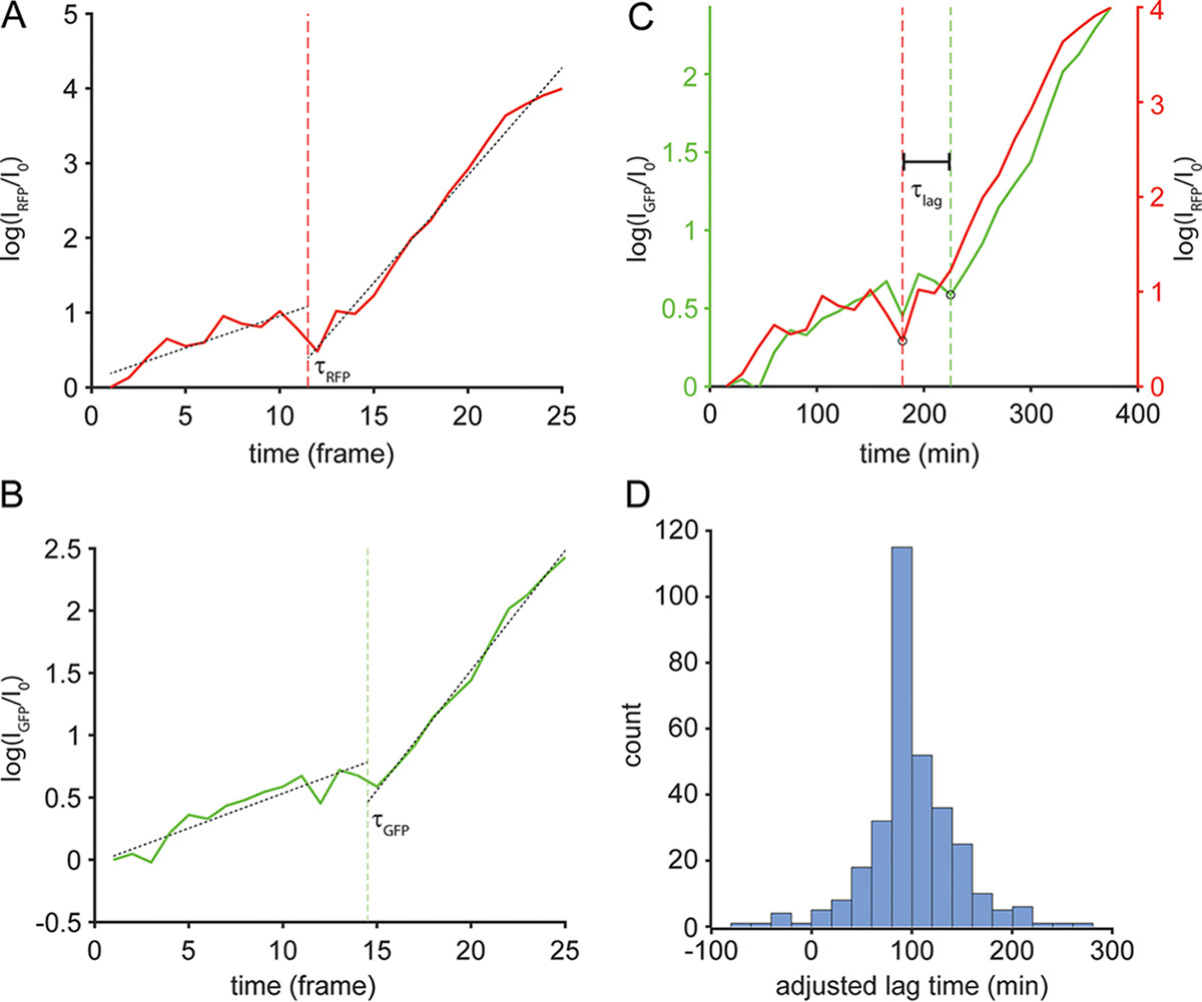
A stereotypical delay relates ExsA and T3SS expression. Change-point analysis was applied to the log-fold change of normalized RFP and GFP trajectories of mixed colony cells that became T3SS-ON by *t* = 6 h (*n* = 6,466 trajectories). (A and B) An example of change point identification for a single cell’s RFP (A) and GFP (B) channels (τ_RFP_ and τ_GFP_). (C) RFP change points were subtracted from GFP change points to obtain lag times (τ_LAG_). (D) Distribution of lag times adjusted for fluorophore maturation times (Fig. S4); the mean ± SD lag time is 89 ± 2.9 min.

In time-lapse images of dual-reporter mixed microcolonies, bacteria show *de novo* RFP and GFP fluorescence during growth on T3SS-activating agarose pads. We observed that an increase in RFP MFI—a marker of ExsA transcription/translation—consistently preceded the increase in GFP MFI—a marker of T3SS gene induction—within any given cell’s lineage and decided to investigate this relationship further. Time-lapse series of 19 mixed phenotype microcolonies of the PA14 dual reporter P_exoT_-sfGFP/*exsA-*RBS*-mTagRFP-t* strain were lineage tracked for 6 h after inoculation to MinS-NTA agarose pads. MFI values within each lineage were normalized by the initial GFP or RFP MFI value measured for the mother cell, yielding the fold change in fluorescence intensity as a function of time. Change point analysis was applied to the normalized GFP and RFP trajectories of each cell that crossed the fluorescence threshold defining T3SS-ON by the end of the 6-h experiment. We determined the times at which the RFP and GFP trajectories showed a significant inflection and plotted the difference in those times for each cell lineage (Fig. 4A to C). The RFP fluorescence inflection point occurred on average 1.2 frames (18 ± 2.9 min) earlier than that for GFP fluorescence (two-tailed Student’s *t* test, *P* < 10^−12^). mTagRFP-t matures more slowly than sfGFP when expressed in E. coli (34); we thought it likely that this would also be the case in P. aeruginosa. In order to determine the mean delay between expression of ExsA/mTagRFP-t and sfGFP, we cloned each fluorescent protein’s gene under the control of an inducible *tac* promoter and measured maturation time in P. aeruginosa following the method of Hebisch et al., in which fluorescence intensity is monitored after the addition of chloramphenicol to stop *de novo* protein translation (35). We calculated the maturation time for sfGFP to be 15.3 min (confidence interval [CI], 10 to 32 min) and that for mTagRFP-t to be 87.1 min (CI, 72.8 to 108 min) (Fig. S4). When these sfGFP and mTagRFP-t maturation times are taken into account, the mean lag time between ExsA and P*_exoT_* expression is ∼89 min (Fig. 4D). This delay may reflect molecular events that occur between initial ExsA expression (e.g., expression of T3SS proteins necessary to assemble a secretion-competent apparatus) and the full upregulation of T3SS expression once ExsE is secreted and ExsD inhibition of ExsA is relieved.

10.1128/mBio.00831-21.6 FIG S4 Calculating maturation times of sfGFP and mTagRFP-t in P. aeruginosa PA14. (A and B) GFP (A) and RFP (B) fluorescence intensity were monitored after the addition of Cm (500 μg/ml; *t* = 0) to exponentially growing PA14 expressing sfGFP (A) or mTagRFP-t (B) from an IPTG-inducible plasmid. Fluorescence intensity (FI) at the time of Cm addition to cultures was set to 0, and values were normalized to the maximal FI reached by samples. The curve-fitting function of MATLAB was used to fit *y* = *a* · (1-exp[−b · x]) to averaged FI data (*n* = 12). Figures are representative of 3 to 5 independent experiments. For GFP, *b* = 0.0654 (95% CI, 0.0309, 0.1); R^2^ = 0.585. For RFP, *b* = 0.0115 (95% CI, 0.00923, 0.0137); R^2^ = 0.962. Download FIG S4, PDF file, 0.1 MB. Copyright © 2021 Lin et al. 2021 Lin et al. https://creativecommons.org/licenses/by/4.0/ This content is distributed under the terms of the Creative Commons Attribution 4.0 International license.

### T3SS-expressing cells divide more slowly.

**FIG 5:**
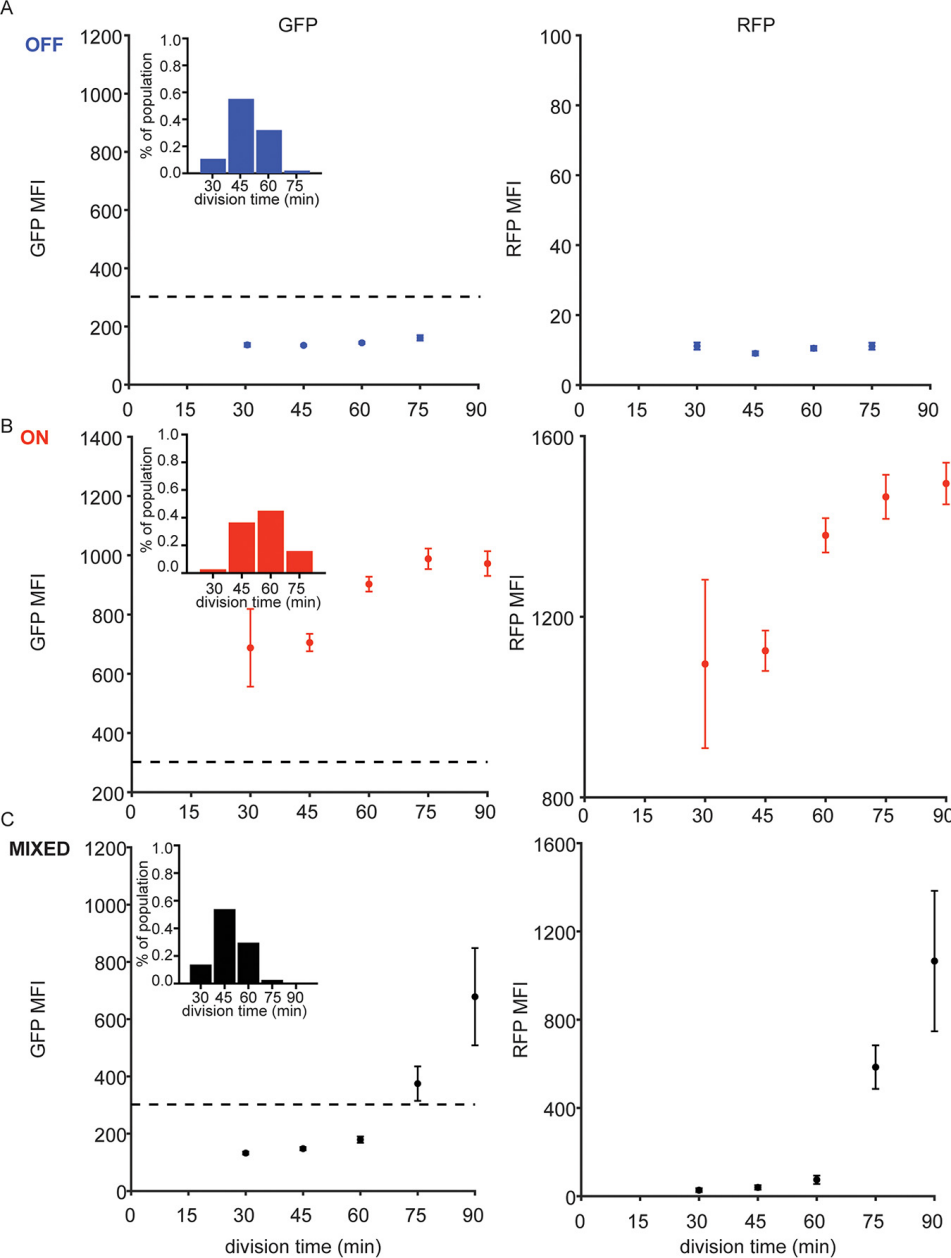
T3SS expression decreases P. aeruginosa fitness. PA14 P_exoT_-sfGFP/*exsA-mTagRFP-t* were lineage traced over 6 h following exposure to T3SS activating signal. Division time was defined as the time from observed birth frame to division frame, excluding cells present in the first and last frames of a time-lapse series. The median RFP and GFP MFI were determined for each cell between its birth and division. (A to C) The distribution of median RFP and GFP MFI as a function of division time is plotted for cells in (A) OFF microcolonies (*n* = 617 events), (B) ON microcolonies (*n* = 394 events), and (C) mixed microcolonies (*n* = 2,492 events). Points show the mean ± SD of MFI for cells with that division time; the dotted line in the GFP MFI plots shows the threshold used to define T3SS-ON cells. For each panel, histogram insets show the distribution of division times (min) for T3SS-OFF, T3SS-ON, and mixed cells.

In *S.* Typhimurium, T3SS expression carries a fitness cost *in vitro* (36), an observation that was extrapolated to the growth of T3SS-expressing cells *in vivo* (8). In our time-lapse experiments, we observed that ON microcolonies contained fewer cells (median, 38; range, 24 to 65) after 6 h of growth on T3SS-activating agarose pads than either OFF (median, 64; range, 30 to 123) or mixed colonies (median, 63; range, 50 to 128). We hypothesized that this might reflect a longer time to division in cells expressing T3SS genes and tested this by directly measuring the duration of a cell’s lifetime—from birth to the following cell division—as a function of its median GFP and RFP intensity during that time. This analysis was carried out for all mixed microcolonies, in which both T3SS-OFF and T3SS-ON cells were present for lineage tracing (Fig. 5C). A total of 2,492 individual cells’ lifetimes were measured; the initial mother cell and all cells present in the final time-lapse frame were excluded since their full lifetimes could not be traced to completion. We observed that the longest lifetimes (75 or 90 min) were measured for cells with the highest GFP MFI, while shorter lifetimes (30 to 60 min) were measured for cells whose GFP MFI was below the threshold for “T3SS-ON.” A similar trend was observed for RFP MFI, with the brightest cells also being those with the longest lifetimes.

We also performed lineage tracking for all cells within 5 ON and 5 OFF microcolonies, again measuring time to division and mean GFP and RFP MFIs for each cell. The distribution of division times differed between ON microcolonies (60.8% of cells with division times of ≥60 min) and OFF microcolonies (34.1% of cells with division times of ≥60 min) (Fig. 5A and B). We confirmed that the introduction of the fluorescent reporters did not slow growth of the parental PA14 wild-type (WT) strain in T3SS-activating media (Fig. S5), suggesting that the single-cell growth differences measured on agarose pads reflect the behavior of reporter-less T3SS-ON and T3SS-OFF cells. We also observed slower growth of Δ*exsD* bacterial cultures, in which T3SS expression is constitutive, than that of T3SS-negative Δ*exsA* cultures in the absence of a T3SS activating signal, again correlating T3SS expression with slower growth (Fig. S5).

10.1128/mBio.00831-21.7 FIG S5 Growth curves of bacterial strains with and without fluorescent reporters. (A to C) Growth of PA14 WT (A), PA14 Δ*exsA* (B), and PA14 Δ*exsD* (C) strains with or without the P_exoT_-sfGFP reporter was followed by measuring OD_600_. The PA14 WT P_exoT_-sfGFP/*exsA-RBS-mTagRFP-t* strain was also tested against the other two PA14 WT strains. Overnight cultures of all strains were diluted 1:1,000 in triplicate into MinS + 10 mM NTA. Cultures were grown in Corning 96-well flat-bottomed plates at 37°C for 24 h in a Tecan M200 plate reader with shaking. Optical density (600 nm) was measured every 12 minutes. (D) PA14 Δ*exsA* and Δ*exsD* overnight cultures were diluted 1:1,000 into MinS medium and grown (*n* = 6) in a 96-well flat-bottomed plate at 37°C for 24 h in a Tecan M200 plate reader with shaking. The optical density (600 nm) was measured every 5 min. All graphs show the mean ± SD at each time point. Download FIG S5, PDF file, 2.6 MB. Copyright © 2021 Lin et al. 2021 Lin et al. https://creativecommons.org/licenses/by/4.0/ This content is distributed under the terms of the Creative Commons Attribution 4.0 International license.

### The majority of T3SS-expressing cells arise from primed cells.

T3SS-ON cells divide more slowly, indicating that T3SS expression is correlated with a fitness cost. Our analysis of mixed microcolonies, with cells whose sequential acquisition of RFP and GFP fluorescence we observed, also indicates that a significant amount of time elapses between *de novo* expression of ExsA and strong induction of T3SS genes. We therefore asked whether the existence of a primed subpopulation allows P. aeruginosa to rapidly generate T3SS-ON cells in response to an activating signal while maintaining a reservoir of more rapidly dividing T3SS-OFF cells that continue to stochastically generate primed cells. To answer this question, we measured the number and phenotype (T3SS-ON or -OFF) of cells at 2, 4, and 6 h of growth in T3SS-activating conditions, over 99 time-lapse movies. For this analysis, “primed” mother cells were defined as those whose RFP MFI was greater than the stepwise cutoff of 140 (AU) at time 0 that separated mother cells yielding ON versus OFF colonies (Fig. 3C).

**FIG 6:**
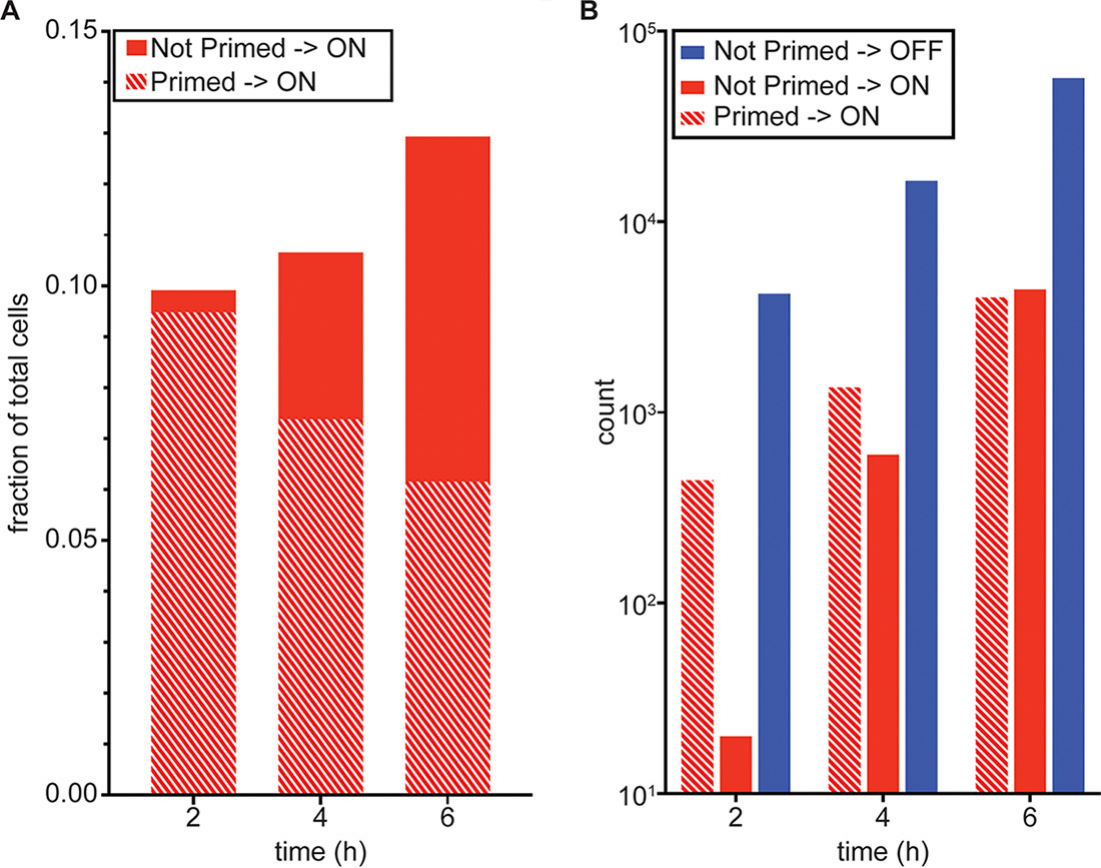
T3SS-ON cells initially arise from primed mother cells. PA14 P_exoT_-sfGFP/*exsA- mTagRFP-t* cells inoculated to T3SS-activating agarose pads were defined as “primed” or “not primed” based on an RFP MFI of >140 AU or <140 AU, respectively, at *t* = 0 h (see Fig. 3C). The GFP MFI for descendants of each mother cell was measured at 2 h, 4 h, and 6 h, allowing descendants to be classified as T3SS-OFF (“OFF”) or T3SS-ON (“ON”) at each of these times. (A) Fraction of total cells classified as “not primed” → T3SS-ON versus “primed” → T3SS-ON. (B) Number of cells classified as “not primed” → T3SS-OFF, “not primed” → T3SS-ON or “primed” → T3SS-ON at each time point (*n* = 6,502 cells in 99 microcolonies).

At hour 0, only 9.3% of the population met this RFP-based definition of a primed cell; these cells also had GFP MFIs that were below the T3SS-ON/OFF threshold. At hour 2 and hour 4, the descendants of this primed subpopulation made up 95.6% and 69.2% of the total T3SS-ON population (Fig. 6). By hour 6, the progeny of the primed cells made up only 6.15% of the total population but accounted for 47.6% of all T3SS-ON cells. The remainder of the T3SS-ON cells were descended from cells that expressed ExsA *de novo*; their progeny made up an increasingly greater proportion of the T3SS-ON population at later times (52.4% by hour 6). Thus, primed cells, though a small fraction of the initial population, generated most of the cells that could quickly express T3SS genes upon exposure to an activating signal.

## DISCUSSION

The nonuniform expression of virulence factors by pathogenic bacteria, whose collaborative activities result in successful infection, has been referred to as “cooperative virulence” (6). When their fitness cost is high, cooperative behaviors can be exploited by cheaters, i.e., individuals who benefit from these shared resources or behaviors without bearing the cost of their production (4). Such a dynamic has been demonstrated for *S.* Typhimurium, whose SP-1 T3SS provokes an inflammatory response in the infected mammalian gut that benefits both SP-1-expressing bacteria and faster growing nonexpressors (8). The emergence of SP-1 mutants—“cheaters”—limits the success of acute infection (8) and prevents transmission to and infection of new hosts (37). In contrast, heterogeneous expression of the SP-1 T3SS by wild-type bacteria maintains a reservoir of genetically wild-type but phenotypically avirulent individuals that benefit from the shared public good provided by SP-1-expressing cells, grow rapidly enough to prevent the population from being overtaken by SP-1-deficient mutants, and retain the ability to express SP-1 T3SS proteins necessary for successful infection and transmission.

In this study, we examined P. aeruginosa, another Gram-negative pathogen that depends on T3SS-dependent virulence for successful acute lung infection (38). T3SS effector-mediated activities, particularly those associated with the phospholipase A_2_ cytotoxin ExoU, can benefit not only coinfecting T3SS-mutant P. aeruginosa (14) but even avirulent E. coli XL-1 Blue (39). P. aeruginosa’s expression of T3SS proteins has been observed to be heterogeneous by multiple investigators, raising the possibility that P. aeruginosa, like *S.* Typhimurium, balances the fitness costs and benefits of T3SS production between phenotypically T3SS-ON and -OFF bacteria. By using single-cell methods that allowed lineage-tracking of T3SS expression over multiple generations, we demonstrated that a clonal P. aeruginosa population indeed gave rise to both T3SS-expressing and -nonexpressing individuals when exposed to a uniform T3SS-activating signal. Our experiments also unequivocally associated T3SS-expression with a fitness cost, with longer division times measured for individual T3SS-ON cells than for T3SS-OFF kin (Fig. 5). We speculate, as have others for Salmonella (36, 37, 40), that slower growth reflects the metabolic costs associated with expressing the abundant structural and effector proteins of the T3SS. This contrasts with the behavior of Yersinia enterocolitica, where phenotypically homogeneous T3SS expression by members of a population is associated with a rapid yet reversible arrest of cell division (41, 42).

P. aeruginosa cells showed three distinct behaviors when shifted from nonactivating to activating conditions. Some cells began expressing sfGFP quickly, yielding microcolonies that were uniformly T3SS-ON (Fig. 2); others went through one or more divisions before a daughter cell and her progeny became T3SS-ON, while many more cells remained T3SS-OFF for the duration of our 6- to 8-h time-lapse experiments. We hypothesized that the cells that quickly responded to activating signals were “primed” to do so by virtue of prior expression of the T3SS transcriptional activator ExsA and assembly of a secretion-component type 3 needle (Fig. 1). In this model, the activating signal would gate open the T3SS needle; ExsE would be secreted; ExsA repression would be relieved; and T3SS gene expression could proceed. In order to search for “primed” cells, we constructed a second chromosomal reporter that coupled ExsA transcription and translation with mTagRFP-t expression. It allowed us to identify a subpopulation of bacteria that expressed ExsA when grown in nonactivating conditions and to demonstrate that these RFP+ cells were uniquely capable of rapid T3SS gene expression when shifted to activating conditions (Fig. 3). This dual reporter also allowed us to see *de novo* expression of ExsA and mTagRFP-t in cells growing under activating conditions and to measure the delay before T3SS gene expression was detected, which we found to be ∼89 min (Fig. 4). This delay between the detection of ExsA production and T3SS gene expression may reflect the time required to transcribe, translate, and assemble a T3SS apparatus; we were not able to develop a method for visualizing T3SS needles in live cells to test this hypothesis explicitly. This delay is consistent, however, with the disproportionate contribution made by primed cells to the T3SS-ON population following the shift to activating conditions (Fig. 6).

Although P. aeruginosa and *S.* Typhimurium (36, 43) demonstrate bimodal expression of T3SS genes when activated, this is not the case for all pathogens. Enteropathogenic E. coli (EPEC) exposed to T3SS-activating conditions uniformly upregulates expression of the Ler master regulator and the T3SS genes under its control (44). When shifted to nonactivating conditions, most EPEC cells stop expressing T3SS genes. A slower-growing subset of the population maintains a T3SS-ON phenotype for many generations, however, generating a bimodal population of T3SS-ON and -OFF individuals. This hysteretic switch is mediated by the *per* operon, whose proteins (PerABC) are expressed bimodally when EPEC experiences activating conditions (44). Only *per*-ON cells maintain T3SS expression after a shift to nonactivating conditions, resulting in a subset of the population that is uniquely hypervirulent toward mammalian cells.

Our work demonstrates that heterogeneous expression of ExsA before bacteria are shifted to activating conditions gives rise to heterogeneous expression of T3SS genes upon activation. It does not address how ExsA heterogeneity arises and whether this process is stochastic or regulated (45). The fact that we observe T3SS heterogeneity for multiple strains of P. aeruginosa, albeit at different ratios of ON versus OFF individuals, suggests that the many described regulators of ExsA transcription and translation probably influence the likelihood that a cell will become primed (23); our dual reporter system will allow us to test this hypothesis in future experiments. Marsden et al. have already demonstrated that mutation of the P*exsA* promoter, which lies directly upstream of *exsA* and is activated by the cAMP-responsive regulator Vfr, dramatically reduces the proportion of bacteria expressing a T3SS GFP transcriptional reporter (16). More work will be needed to learn whether expression of *exsA* from this promoter is required to generate the “primed” phenotype and whether intracellular concentrations of cAMP can tune the proportion of primed cells.

## MATERIALS AND METHODS

### Bacterial strains and growth conditions.

All strains and plasmids used in this study are listed in Table S1. Escherichia coli was cultured in Luria broth (LB), and Pseudomonas aeruginosa was cultured in LB or Vogel-Bonner minimal (VBM) medium (46) with antibiotics as required at the following concentrations: gentamicin, 15 μg ml^−1^ for E. coli or 100 μg ml^−1^ for P. aeruginosa; ampicillin, 100 μg ml^−1^ for E. coli; kanamycin, 50 μg ml^−1^ for E. coli; carbenicillin, 200 μg ml^−1^ for P. aeruginosa; tetracycline, 20 μg ml^−1^ for E. coli or 100 μg ml^−1^ for P. aeruginosa. All bacterial strains were maintained at −80°C as 15% (vol/vol) glycerol stocks and were freshly plated to LB or VBM agar prior to each experiment.

10.1128/mBio.00831-21.1 TABLE S1 Bacterial strains and plasmids used in this study. Download Table S1, PDF file, 0.2 MB. Copyright © 2021 Lin et al. 2021 Lin et al. https://creativecommons.org/licenses/by/4.0/ This content is distributed under the terms of the Creative Commons Attribution 4.0 International license.

To induce T3SS expression, P. aeruginosa was grown in MinS medium (25 mM KH_2_PO_4_, 0.1 M NH_4_Cl, 90 mM C_4_H_4_O_4_Na_2_.6H_2_O, 50 mM C_5_H_8_NO_4_Na, 2.5% [vol/vol] glycerol, 5 mM MgSO_4_, 18 μM FeSO_4_.7H_2_O) plus 10 mM nitriloacetate (NTA) at 37°C with aeration. To suppress T3SS expression in minimal medium, P. aeruginosa was grown in MinS medium plus 5 mM CaCl_2_ at 37°C with aeration (33).

### Plasmid and strain construction.

PA14 deletion mutants for *exsA* and *exsD* were constructed as previously described (47, 48). Briefly, primers were designed to amplify ∼1-kb regions flanking the genes of interest; these fragments were joined by overlap extension PCR (Table S2). This knockout (KO) allele was integrated into the Gateway-adapted pDONRX plasmid (49), and the construction was confirmed by Sanger sequencing (Keck Sequencing Facility, Yale). Biparental mating was carried out on low-salt LB agar plates (containing 5 g/liter NaCl), and merodiploids were selected on VBM-gentamicin plates. Counterselection on VBM plates containing 10% (wt/vol) sucrose allowed identification of recombination events that led to plasmid backbone loss; these were screened by PCR to identify Δ*exsA* and Δ*exsD* clones.

10.1128/mBio.00831-21.2 TABLE S2 Primers used in this study. Download Table S2, PDF file, 0.1 MB. Copyright © 2021 Lin et al. 2021 Lin et al. https://creativecommons.org/licenses/by/4.0/ This content is distributed under the terms of the Creative Commons Attribution 4.0 International license.

An *exsA*-RBS-*mTagRFP-t*-*exsD* construct was designed to couple mTagRFP-t (50) transcription and translation with that of ExsA. The *exsD* promoter, which is embedded within the *exsA* gene, was reconstructed 3′ to mTagRFP-t. This cassette was synthesized by Genewiz and amplified with *attB1* and *attB2* site-containing flanking primers and cloned into pDONRX. Biparental mating and Sanger sequencing confirmation were performed as described.

The P_exoT_-superfolder GFP (sfGFP) reporter was constructed by PCR amplifying the *exoT* promoter region (51) from PA14 genomic DNA with primers P*exoT*-F and P*exoT-*R and cloning it in place of the *exoU* promoter in the previously described P*_exoU_*-sfGFP reporter (14). The construct was integrated into the P. aeruginosa chromosome at the *attB* site in the strains listed in Table S1. Vector backbone sequences were excised as previously described (52). Chemically competent E. coli DH5α (Invitrogen) was used for transformations and plasmid purification, and S17.1 (53) was use for mating with P. aeruginosa.

### Flow cytometry.

Single bacterial colonies carrying the P*_exoU_*-sfGFP reporter were inoculated into LB and grown overnight with aeration at 37°C, rinsed with MinS, and subcultured into MinS + 10 mM NTA to induce T3SS expression. Culture aliquots were sampled at 16 h and 24 h postinduction and processed for flow cytometry as previously described (14). Briefly, cells fixed with 1% paraformaldehyde were analyzed for GFP emission at 530 nm using an LSRII flow cytometer (BD Biosciences). Bacteria were identified by side scatter. At least 70,000 events were analyzed using FlowJo (v. 9.7.1) software to determine P*_exoU_*-sfGFP expression. GFP-positive and -negative gates were set using PA103Δ*exsA* PexoU-sfGFP so that the mean fluorescence intensity of events scored as GFP-positive exceeded that observed for 99% of Δ*exsA* bacteria.

### Fluorescence microscopy and time point/time-lapse imaging.

For time point experiments, single bacterial colonies were inoculated into LB, grown overnight with aeration at 37°C, rinsed with MinS, and subcultured into MinS + 10 mM NTA or MinS + 5 mM CaCl_2_. Pads of 1% (wt/vol) gellan gum were made by pipetting 200 μl between two glass slides separated by several tape layers. At hours 0, 4, 8, and 12, 2 μl of subculture was pipetted onto pads and sealed with a coverslip and clear nail polish prior to image acquisition.

For time-lapse experiments, single bacterial colonies were inoculated into LB, grown overnight with aeration at 37°C, and then subcultured 1:30 into MinS + 5 mM CaCl_2_ medium. MinS + 10 mM NTA 1.5% (wt/vol) agarose pads were prepared as previously described (54). Briefly, 0.15 g of low-melt agarose was slowly melted in 10 ml of MinS + 10 mM NTA medium. One ml of mixture was sandwiched between 22-mm^2^ coverslips and allowed to dry for at least 1 h. At an optical density at 600 nm (OD_600_) of ∼0.4 to 0.6, 500 μl of subculture was pelleted, washed with 500 μl MinS-only medium, and then diluted to an OD_600_ of 0.01. Then, 2 μl of the diluted culture was spotted onto MinS + 10 mM NTA agarose pads, which were left to dry at room temperature for 15 to 20 min. The pads were then flipped onto Mattek 35 mm uncoated glass bottom dishes (no. 1.5 coverslip, 20 mm diameter), and imaging was initiated after ∼15 min microscopy setup.

Fluorescence microscopy was performed with a Nikon Eclipse T*i* microscope equipped with a phase-contrast Nikon Plan Apo λ 100×/NA 1.45 oil objective and Lumencor SOLA SE light engine. Images were captured with an Andor Zyla VSC-01400 camera in black and white, false-colored, and superimposed using NIS Elements (version 4.50) software. GFP channel images were captured using the Chroma enhanced GFP (eGFP) single-band filter set for an exposure time of 3 s, and RFP channel images were captured with the Chroma mCherry, TexasRed filter set for an exposure time of 2 s, both with the ND8 filter engaged. Early time point exposures were set at 10 s for both GFP and RFP channels.

### Image processing and data analysis.

A customized semiautomated pipeline was used to analyze raw microscopy images. First, images containing single colonies were saved as .TIF files in Fiji (55); for each colony, individual cells were segmented and tracked in a modified version of Oufti (56, 57) based on the phase-contrast channel, with manual user supervision. A series of custom routines, available at https://github.com/caseygrun/oufti in MATLAB2018 (Mathworks, Natick, MA), were then used to calculate the mean fluorescence intensity (MFI) and cell division time. For each cell at each time frame, MFI was calculated by averaging the raw per-pixel fluorescence intensity over the specific cell’s mask and subtracting the background fluorescence level. To classify cells as ON or OFF, MFIs were calculated for both positive (Δ*exsD*) and negative (Δ*exsA*) controls as described. For each of these two populations, a Gaussian distribution was fitted using a maximum likelihood estimator of mean (μ) and standard deviation (σ). A decision boundary, defined as the intensity x*, was defined as the intensity where an MFI would be equally likely to be drawn from either control, i.e., where the likelihood ratio *L* was 1:

$$L\left(x^{*}\right)=\frac{\sigma_{\Delta \mathrm{exsD}}\exp\left(\frac{-{\left(x^{*}-\mu_{\Delta \mathrm{exsA}}\right)}^{2}}{2\sigma_{\Delta \mathrm{exsA}}^{2}}\right)}{\sigma_{\Delta \mathrm{exsA}}\exp\left(\frac{-{\left(x^{*}-\mu_{\Delta \mathrm{exsD}}\right)}^{2}}{2\sigma_{\Delta \mathrm{exsD}}^{2}}\right)}=1$$

Upper and lower confidence intervals for x* were formed by using the respective 5% and 95% probability thresholds based on the *χ*^2^ *P* value derived from the likelihood ratio test statistic, λ = −2log *L*. The decision boundary was calculated independently for each time point and applied to colonies to classify them as ON, OFF, or mixed, based on the percentage of cells falling above or below the threshold. Intensity was adjusted for microscope settings based on an internal calibration as necessary for each experiment.

To track individual cells’ behavior, MFI trajectories were then generated by following cell lineages within individual colonies and considering their MFI trajectories in time. Division time was calculated by counting the number of frames between a cell’s first and last appearance, inclusive; cells that appeared in the first frame of the movie were discarded. To characterize the dynamics of individual cell trajectories, change point analysis (findchangepts MATLAB function) was applied to locate the time point such that the signal had the most stable (i.e., lowest square error) slope and mean before and after (58). We applied this routine to both GFP and RFP signals to extract timescales for individual cell fluorescence trajectories and compared these timescales on a cell-ensemble level to calculate the delay between *exsA* and *exsD* expression. All plots were generated either directly in MATLAB or from data sets exported to Prism GraphPad 7.

### Fluorescent protein maturation time estimation.

The sfGFP gene was amplified from the miniCTX2-P*_exoU_*-sfGFP plasmid (14), and the mTagRFP-t gene was amplified from pUC57/*exsA*-RBS-*mTagRFP-t* (Tables S1 and S2). Both amplicons included the associated RBS and were digested with EcoRI and HindIII and then ligated into pMMB67EH (59) with T4 ligase. The ligation reaction was transformed into chemically competent DH5α and grown on LB plates with ampicillin (100 μg ml^−1^). DH5α was grown overnight in Luria broth supplemented with 24 g yeast extract (LB24) (60) to increase plasmid yield. The purified plasmid was transformed into chemically competent PA14 and selected for on LB plates with carbenicillin (200 μg ml^−1^).

PA14 carrying pMMB-sfGFP or -mTagRFP-t was cultured in LB24 plus 200 μg ml^−1^ carbenicillin (at 37°C, 225 rpm), subcultured 1:100 into M9 plus 1% CAA (Casamino Acids) with 150 μg ml^−1^ carbenicillin, dispensed into a 96-well black plate with flat-bottom clear wells (200 μl/well), and incubated at 37°C in a Tecan Infinite M200 plate reader (12 wells/condition). Absorbance (OD_600_) and fluorescence (Ex/Em) were measured at 2- to 5-min intervals before and after the addition of IPTG (isopropyl-β-d-thiogalactopyranoside; final concentration, 0.5 mM) at early log phase to induce fluorophore expression. After 2 to 3 h, protein synthesis was inhibited in a subset of wells by the addition of chloramphenicol (Cm; final concentration, 500 μM). Background fluorescence was determined from samples grown in the absence of IPTG.

Maturation times for sfGFP and mTagRFP-t were calculated according to reference 35. Briefly, the fluorescence intensity (FI) at the time of Cm addition was set to 0, and the maximum (100%) FI signal was set to 1. Fluorescence data between the time of Cm addition and FI signal saturation were fitted using MATLAB to (*y*) = a* (1 − exp[−*b* · *x*]), where *b* is the characteristic time constant at normalized 63% FI, and 1/*b* is the maturation time.
