# Supplementary material for: A Primed Subpopulation of Bacteria Enables Rapid Expression of the Type 3 Secretion System in Pseudomonas aeruginosa
Source: mBio. 2021 Jun 22;12(3):e00831-21. doi: 10.1128/mBio.00831-21 (PMC8262847; doi:10.1128/mBio.00831-21)
Supplement: FIG S1 [file mbio.00831-21-sf001.pdf]

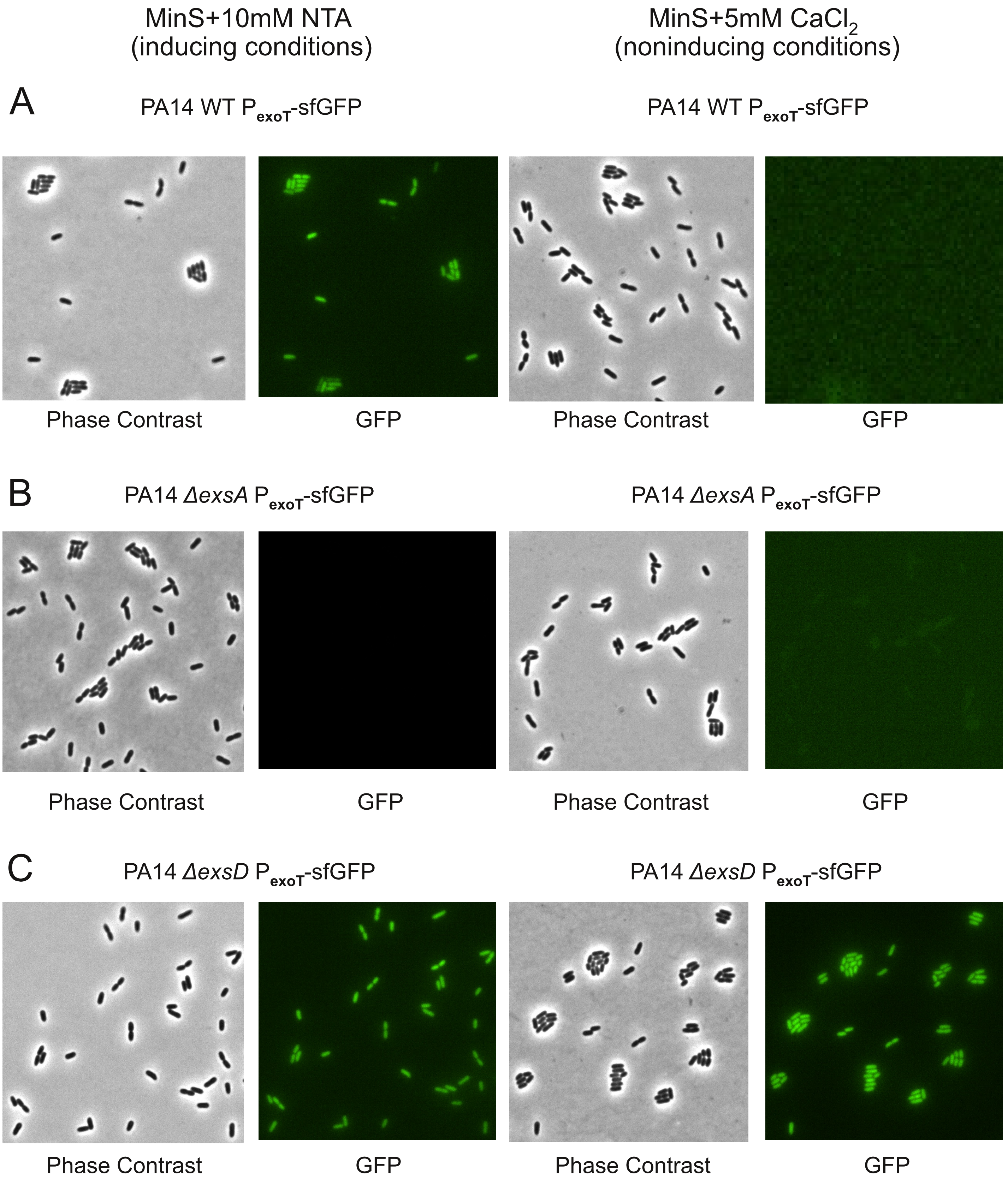

Figure S1. The PexoT-sfGFP reporter responds to extrinsic and intrinsic T3SS-activating signals. (A) PA14 WT, (B) PA14 Δ*exsA*, and (C) PA14 Δ*exsD* with the *attB::P*$_{exoT}$*-sfGFP* reporter were grown in MinS+10mM NTA (T3SS-activating condition) or MinS+5mM $CaCl_2$ (T3SS non-activating condition) as indicated.
