# Supplementary material for: A Primed Subpopulation of Bacteria Enables Rapid Expression of the Type 3 Secretion System in Pseudomonas aeruginosa
Source: mBio. 2021 Jun 22;12(3):e00831-21. doi: 10.1128/mBio.00831-21 (PMC8262847; doi:10.1128/mBio.00831-21)
Supplement: FIG S2 [file mbio.00831-21-sf002.pdf]

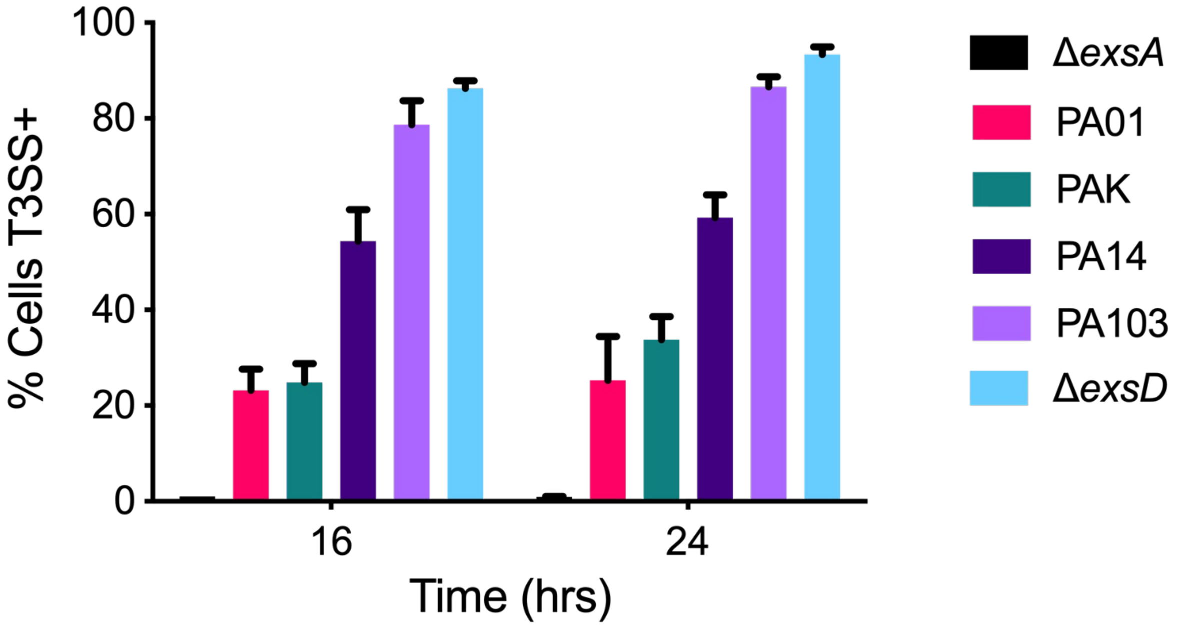

Figure S2. Heterogeneous T3SS is observed in multiple *P. aeruginosa* strain backgrounds. *P. aeruginosa* strains PAO1, PAK, PA14, and PA103 with the *attB::*P_{exoU}*-sfGFP* reporter were grown in MinS+10mM NTA, sampled at the indicated times and analyzed by flow cytometry. PA103Δ*exsA* and PA103Δ*exsD* serve as negative and positive controls, respectively.
