# Supplementary material for: A Primed Subpopulation of Bacteria Enables Rapid Expression of the Type 3 Secretion System in Pseudomonas aeruginosa
Source: mBio. 2021 Jun 22;12(3):e00831-21. doi: 10.1128/mBio.00831-21 (PMC8262847; doi:10.1128/mBio.00831-21)
Supplement: FIG S3 [file mbio.00831-21-sf003.pdf]

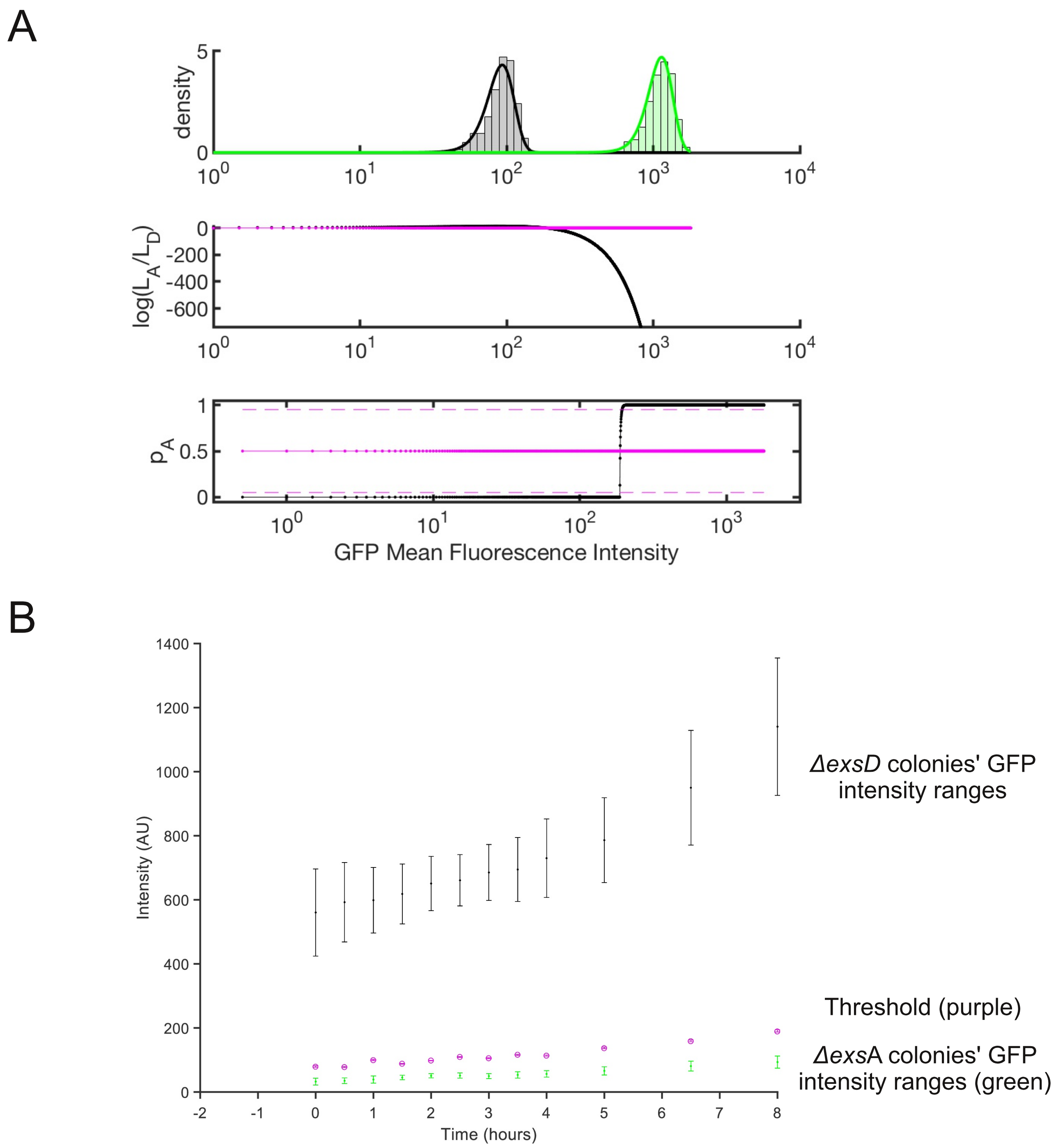

Figure S3. A T3SS-ON threshold is defined using constitutively expressing and non-expressing strains. (A) To define individual cells as either T3SS-ON or T3SS-OFF, positive (PA14 Δ*exsD*) and negative (PA14 Δ*exsA*) controls with the P_{exoT}-sfGFP reporter were grown in the T3SS-activating conditions and tracked with time-lapse microscopy. A decision threshold was calculated using the intensity distributions of the positive and negative controls. Each distribution was fit to a Gaussian and the threshold defined as the intensity at which the likelihood of each Gaussian was equal, i.e., where the log likelihood ratio of the MLE-fit Gaussians was equal to 0. Equivalently, a *p* value could be calculated since the statistic -2 * log(K) (K= likelihood ratio) is drawn from a chi-squared distribution and the threshold was calculated where *p*=0.5 and lower and upper confidence intervals were set where *p*=.05 and 0.95, respectively. A cell with a specific value of GFP MFI above the threshold has a higher likelihood of being T3SS-ON. The decision threshold at Hour 6 is shown. (B) The decision threshold (in purple) graphed over 8 hours with the GFP mean fluorescence intensity ranges for the positive (PA14 Δ*exsD*) and negative (PA14 Δ*exsA*) controls shown in black and green, respectively.
