# Supplementary material for: A Primed Subpopulation of Bacteria Enables Rapid Expression of the Type 3 Secretion System in Pseudomonas aeruginosa
Source: mBio. 2021 Jun 22;12(3):e00831-21. doi: 10.1128/mBio.00831-21 (PMC8262847; doi:10.1128/mBio.00831-21)
Supplement: FIG S4 [file mbio.00831-21-sf004.pdf]

A

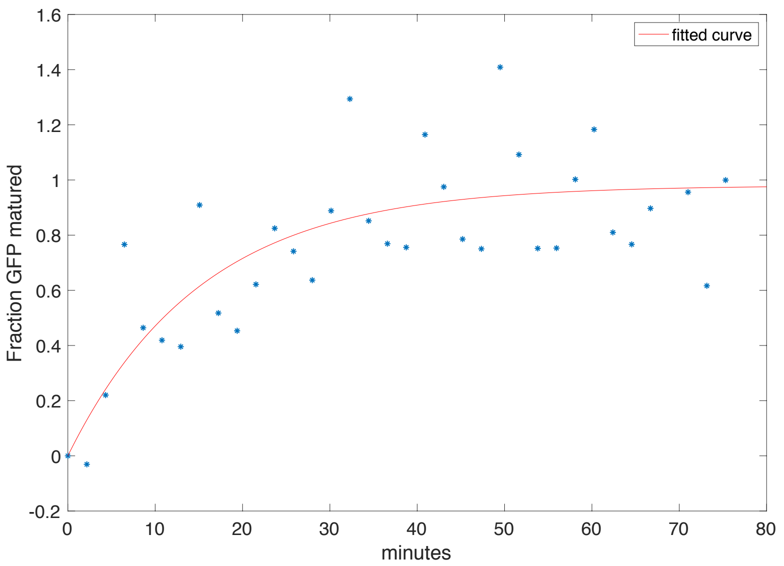

B

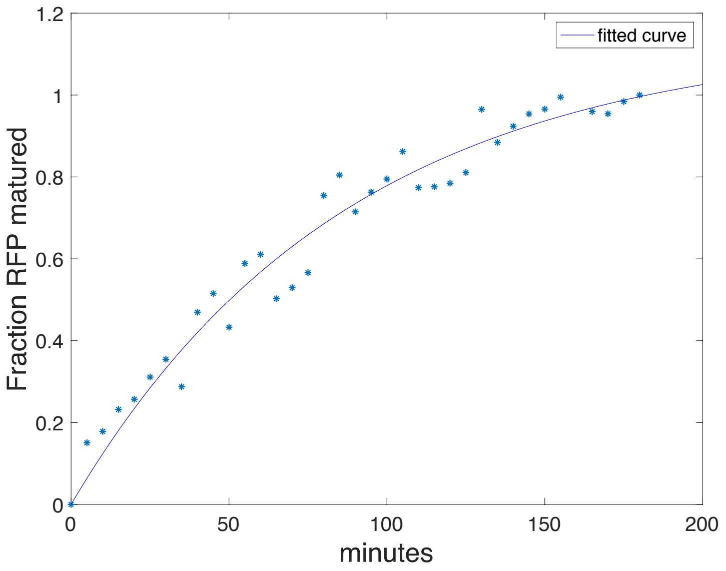

Figure S4. Calculating maturation times of sfGFP and mTagRFP-t in *P. aeruginosa* PA14.
GFP (A) and RFP (B) fluorescence intensity were monitored after the addition of Cm (500 μg/mL; t=0) to exponentially growing PA14 expressing sfGFP (A) or mTagRFP-t (B) from an IPTG-inducible plasmid. Fluorescence intensity (FI) at time of Cm addition to cultures was set to 0, and values were normalized to the maximal FI reached by samples. The curve fitting function of Matlab was used to fit $y = a*(1-\exp(-b*x))$ to averaged FI data (n=12). Figures are representative of 3-5 independent experiments. For GFP, b = 0.0654 (95% CI 0.0309, 0.1); R2= 0.585. For RFP, b = 0.0115 (95% CI 0.00923, 0.0137); R2 = 0.962.
