# Supplementary material for: A Primed Subpopulation of Bacteria Enables Rapid Expression of the Type 3 Secretion System in Pseudomonas aeruginosa
Source: mBio. 2021 Jun 22;12(3):e00831-21. doi: 10.1128/mBio.00831-21 (PMC8262847; doi:10.1128/mBio.00831-21)
Supplement: FIG S5 [file mbio.00831-21-sf005.pdf]

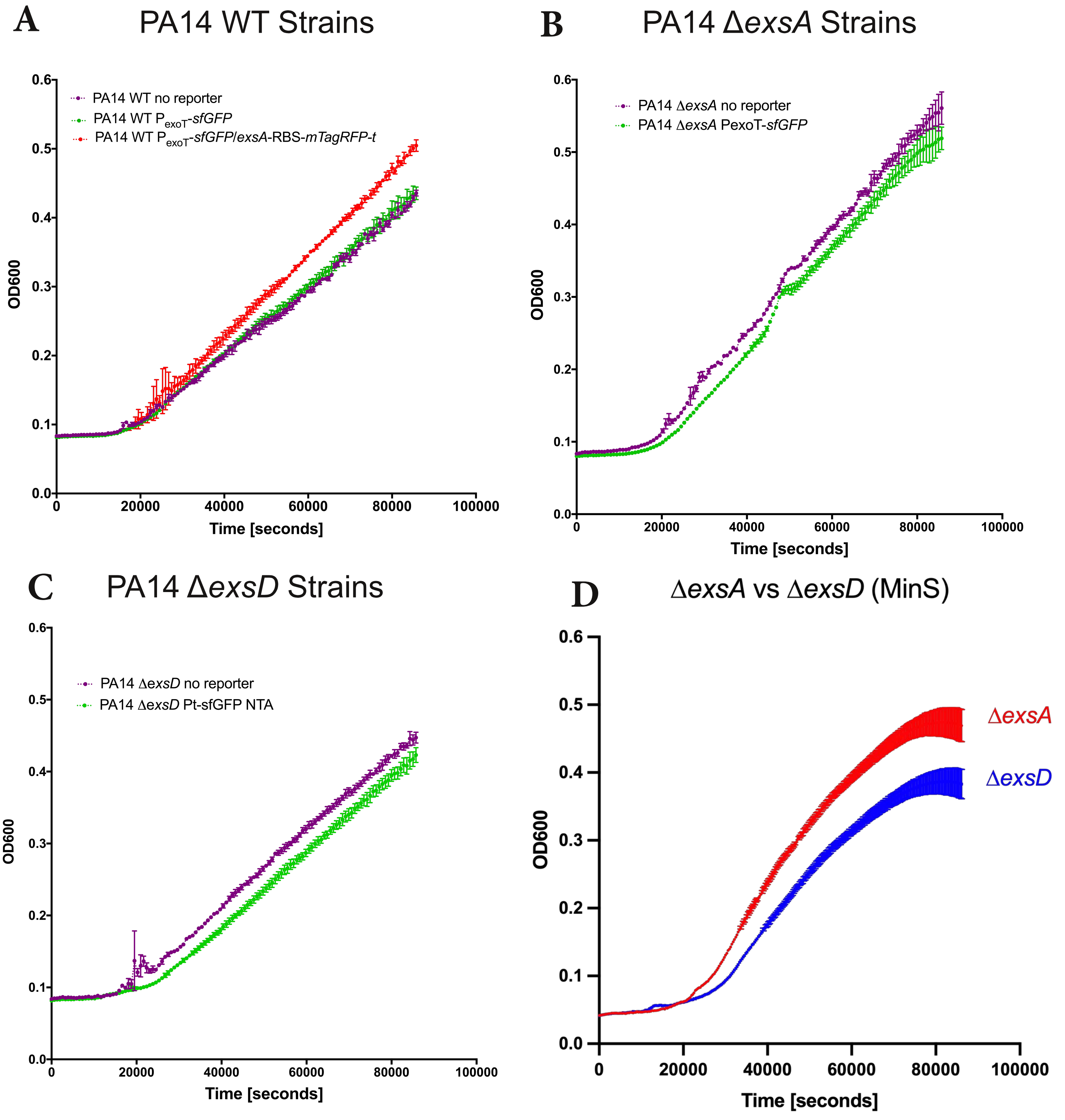

Figure S5. Growth curves of bacterial strains with and without fluorescent reporters. Growth of PA14 WT (A), PA14 Δ*exsA* (B), and PA14 Δ*exsD* (C) strains with or without the P_{exoT}-sfGFP reporter was followed by measuring OD_{600}. The PA14 WT P_{exoT}-sfGFP /exsA-RBS-mTagRFP-t strain was also tested against the other two PA14 WT strains. Overnight cultures of all strains were diluted 1:1000 in triplicate into MinS+10mM NTA. Cultures were grown in Corning 96-well flat-bottomed plates at 37°C for 24 hours in a Tecan M200 plate reader with shaking. Optical density (600nm) was measured every 12 minutes. (D) PA14 Δ*exsA* and Δ*exsD* overnight cultures were diluted 1:1000 into MinS media and grown (n=6) in a 96 well flat-bottomed plate at 37˚C for 24 hours in a Tecan M200 plate reader with shaking. Optical density (600nm) was measured every 5 minutes. All graphs show mean ± S.D. at each time point.
