# Supplementary material for: A Primed Subpopulation of Bacteria Enables Rapid Expression of the Type 3 Secretion System in Pseudomonas aeruginosa
Source: mBio. 2021 Jun 22;12(3):e00831-21. doi: 10.1128/mBio.00831-21 (PMC8262847; doi:10.1128/mBio.00831-21)
Supplement: TABLE S1 [file mbio.00831-21-st001.pdf]

Table S1. Bacterial strains and plasmids used in this study

Strain or plasmid	Description or relevant genotype	Source or reference
E. coli strains		
DH5α	*supE44 ΔlacU169*(Φ80d*lac*ZΔM15)*hsdR17 thi-1 relA1 recA1*; Used for subcloning	Invitrogen
S17.1	*thi pro hsdR recA* RP4-2 (Tc::Mu) (Km::Tn7); Used for mating constructs into *P. aeruginosa*	(1)
P. aeruginosa strains		
PA14	Wild type isolate	F. Ausubel
PA14 *ΔexsA*	Deletion of *exsA*; does not express T3SS	This work
PA14 *ΔexsD*	Deletion of *exsD*; constitutively expresses T3SS	This work
PA14 *attB::*P_{exoT}-*sfGFP*	*sfGFP* under the control of native *exoT* promoter integrated at the *attB* site	This work
PA14 *ΔexsA attB::*P_{exoT}-*sfGFP*	Deletion of *exsA*; *sfGFP* under the control of native *exoT* promoter integrated at the *attB* site	This work
PA14 *ΔexsD attB::*P_{exoT}-*sfGFP*	Deletion of *exsD*; *sfGFP* under the control of native *exoT* promoter integrated at the *attB* site	This work
PA14 *attB::*P_{exoT}-*sfGFP exsA*-RBS-*mTagRFP-t*	*sfGFP* under the control of native *exoT* promoter integrated at the *attB* site*; mTagRFP-t* gene inserted after *exsA* with its own RBS	This work
PA103 *attB*::P_{exoU}-sfGFP	*sfGFP* under the control of native *exoU* promoter integrated at the *attB* site	(3)
PA103ΔexsA *attB*::P_{exoU}-sfGFP	*sfGFP* under the control of native *exoU* promoter integrated at the *attB* site	(3)
PA103ΔexsD *attB*::P_{exoU}-sfGFP	*sfGFP* under the control of native *exoU* promoter integrated at the *attB* site	(3)
PAO1 *attB*::P_{exoU}-sfGFP	*sfGFP* under the control of native *exoU* promoter integrated at the *attB* site	This work
PAK *attB*::P_{exoU}-sfGFP	*sfGFP* under the control of native *exoU* promoter integrated at the *attB* site	This work
PA14 *attB*::P_{exoU}-sfGFP	*sfGFP* under the control of native *exoU* promoter integrated at the *attB* site	This work
Plasmids		
pDONRX	Gateway-adapted suicide vector; Gm^R	(2)
pDONRX /*ΔexsA*	Vector to generate unmarked *exsA* deletion via homologous recombination; Gm^R	This work
pDONRX /*ΔexsD*	Vector to generate unmarked *exsD* deletion via homologous recombination; Gm^R	This work
mini-CTX2 P_{exoU}-sfGFP	Vector to integrate P_{exoU}-sfGFP cassette into *attB* insertion site; Ap^R	(3)
mini-CTX2 P_{exoT}-sfGFP	Vector to integrate P_{exoT}-sfGFP cassette into *attB* insertion site; Tc^R	This work
pFLP2	Used to flip out mini-CTX2 backbone; Ap/Cb^R	(4)
pUC57/*exsA*-RBS-*mTagRFP-t*	Synthesized construct by Genewiz; Km^R	This work
pDONRX/*exsA*-RBS-*mTagRFP-t*	Vector to introduce *exsA*-RBS-*mTagRFP-t* into chromosome by homologous recombination; Gm^R	This work
pMMB67EH	Expression vector with IPTG-inducible *tac* promoter; lacIq+	(5)
pMMB-sfGFP	sfGFP cloned under control of the *tac* promoter	This work
pMMB-mTagRFP-t	mTagRFP-t cloned under control of the *tac* promoter	This work

Tc, tetracycline; Km, kanamycin; Gm, gentamicin; Ap, ampicillin; Cb, carbenicillin.

References

1. Simon R, Priefer U, Puhler A. 1983. A broad host range mobilization system for in vivo genetic engineering: transposon mutagenesis in Gram negative bacteria. Biotechnology 1:784-791.
2. Fulcher NB, Holliday PM, Klem E, Cann MJ, Wolfgang MC. 2010. The *Pseudomonas aeruginosa* Chp chemosensory system regulates intracellular cAMP levels by modulating adenylate cyclase activity. Mol Microbiol 76:889-904.
3. Czechowska K, McKeithen-Mead S, Al Moussawi K, Kazmierczak BI. 2014. Cheating by type 3 secretion system-negative *Pseudomonas aeruginosa* during pulmonary infection. Proc Natl Acad Sci U S A 111:7801-6.
4. Hoang TT, Karkhoff-Schweizer RR, Kutchma AJ, Schweizer HP. 1998. A broad-host-range Flp-FRT recombination system for site-specific excision of chromosomally-located DNA sequences: application for isolation of unmarked *Pseudomonas aeruginosa* mutants. Gene 212:77-86.
5. Furste JP, Pansegrau W, Frank R, Blocker H, Scholz P, Bagdasarian M, Lanka E. 1986. Molecular cloning of the plasmid RP4 primase region in a multi-host range *tacP* expression vector. Gene 48:119-131.
