# Supplementary material for: A Primed Subpopulation of Bacteria Enables Rapid Expression of the Type 3 Secretion System in Pseudomonas aeruginosa
Source: mBio. 2021 Jun 22;12(3):e00831-21. doi: 10.1128/mBio.00831-21 (PMC8262847; doi:10.1128/mBio.00831-21)
Supplement: TABLE S2 [file mbio.00831-21-st002.pdf]

**Table S2. Primers used in this study**

| Purpose | Primer | Sequence (5' - 3') |
|---|---|---|
| **Chromosomal constructs** | | |
| *ΔexsA* | attB1-exsA-KO-forw | GGGGACAAGTTTGTACAAAAAAGCAGGCTCAACGTGGCTGGCGATCCG |
| | exsA-KO-rev | CGTCAGTTATTTTTAGCCCGGCAGCCAAGAGATTTGGCTCCTTGCA |
| | exsA-KO-forw | TGCAAGGAGCCAAATCTCTTGGCTGCCGGGCTAAAAATAACTGACG |
| | attB2-exsA-KO-rev | GGGGACCACTTTGTACAAGAAAGCTGGGTGATGATCCATGATTCCTCGGTA |
| *ΔexsD* | attB1- exsD-KO-forw | GGGGACAAGTTTGTACAAAAAAGCAGGCTCCGTCCAGGACATCGATTCC |
| | exsD-KO-rev | CGCTCAGCTCTGCCAGTAGAAGTCTTCCTGCTCCATTCTCTG |
| | exsD-KO-forw | CAGAGAATGGAGCAGGAAGACTTCTACTGGCAGAGCTGAGCG |
| | attB2-exsD-KO-rev | GGGGACCACTTTGTACAAGAAAGCTGGGTCGCTGCGCAGGGTGTATTGC |
| $P_{exoT}$-*sfGFP* | PexoT-F | GA**GCGGCCGC**TACGCCGTCCGCCATGAGGC |
| | PexoT-R | CA**CCCGGG**GATGTTTCCCCGCCAGTCTAG |
| | sfGFP-R | GAGA**CTCGAG**ACTTATTAGGATCCGCCAGCACCTT |
| **Plasmid constructs** | | |
| pMMB67EH - sfGFP | pMMB67-sfGFP-F | CAGGC**GAATTC***AGGAGG*TGAGAATGTCTAAAGGTGA |
| | pMMB67-sfGFP-R | CCCG**AAGCTT**CTCGAGACTTATTAGGATCCGCCAG |
| pMMB67EH – mTagRFP-t | pMMB67-mTagRFP-t-F | GAGA**GAATTC***AGGAGGA*TACTAATGGTG |
| | pMMB67-mTagRFP-t-R | GAGA**AAGCTT**TCCTTTACTTGTACAGCTC |

Underlined = *attB* site; Bold and underlined = restriction site; Italicized = RBS.
